# Sex differences associated with adverse drug reactions resulting in hospital admissions

**DOI:** 10.1186/s13293-021-00377-0

**Published:** 2021-05-03

**Authors:** L. C. Hendriksen, P. D. van der Linden, A. L. M. Lagro-Janssen, P. M. L. A. van den Bemt, S. J. Siiskonen, M. Teichert, J. G. Kuiper, R. M. C. Herings, B. H. Stricker, L. E. Visser

**Affiliations:** 1 Department of Epidemiology, Erasmus Medical Center, Rotterdam, The Netherlands; 2 Department of Clinical Pharmacy, Tergooi Hospital, Hilversum, The Netherlands; 3 Department of Primary and Community Care, Gender and Women’s Health, Radboud University Medical Centre, Nijmegen, The Netherlands; 4 Department of Clinical Pharmacy and Pharmacology, University Medical Center Groningen, Groningen, The Netherlands; 5 Division of Pharmacoepidemiology & Clinical Pharmacology, Utrecht Institute for Pharmaceutical Sciences, Faculty of Science, Utrecht University, Utrecht, The Netherlands; 6 Doctoral Programme in Population Health, Faculty of Pharmacy, University of Helsinki, Helsinki, Finland; 7 Department of Clinical Pharmacy & Toxicology, Leiden University Medical Center, Leiden, The Netherlands; 8 PHARMO Institute for Drug Outcomes Research, Utrecht, The Netherlands; 9 Department of Epidemiology and Data Science, Amsterdam UMC, location VUmc, Amsterdam, The Netherlands; 10 Department of Clinical Pharmacy, Haga Teaching Hospital, The Hague, The Netherlands; 11 Department of Hospital Pharmacy, Erasmus Medical Center, Rotterdam, The Netherlands

**Keywords:** Sex differences, Adverse drug reactions, Hospital admissions, Pharmacoepidemiology

## Abstract

**Background:**

Adverse drug events, including adverse drug reactions (ADRs), are responsible for approximately 5% of unplanned hospital admissions: a major health concern. Women are 1.5–1.7 times more likely to develop ADRs. The main objective was to identify sex differences in the types and number of ADRs leading to hospital admission.

**Methods:**

ADR-related hospital admissions between 2005 and 2017 were identified from the PHARMO Database Network using hospital discharge diagnoses. Patients aged ≥ 16 years with a drug possibly responsible for the ADR and dispensed within 3 months before admission were included. Age-adjusted odds ratios (OR) with 95% CIs for drug-ADR combinations for women versus men were calculated.

**Results:**

A total of 18,469 ADR-related hospital admissions involving women (0.35% of all women admitted) and 14,678 admissions involving men (0.35% of all men admitted) were included. Most substantial differences were seen in ADRs due to anticoagulants and diuretics. Anticoagulants showed a lower risk of admission with persistent haematuria (ORadj 0.31; 95%CI 0.21, 0.45) haemoptysis (ORadj 0.47, 95%CI 0.30,0.74) and subdural haemorrhage (ORadj 0.61; 95%CI 0.42,0.88) in women than in men and a higher risk of rectal bleeding in women (ORadj 1.48; 95%CI 1.04,2.11). Also, there was a higher risk of admission in women using thiazide diuretics causing hypokalaemia (ORadj 3.03; 95%CI 1.58, 5.79) and hyponatraemia (ORadj 3.33, 95%CI 2.31, 4.81) than in men.

**Conclusions:**

There are sex-related differences in the risk of hospital admission in specific drug-ADR combinations. The most substantial differences were due to anticoagulants and diuretics.

**Supplementary Information:**

The online version contains supplementary material available at 10.1186/s13293-021-00377-0.

## Background

Interest for sex differences in drug use and its effects is increasing. This is partly explained by a different incidence of several diseases in women compared with men. For example, women are more often affected by migraine and autoimmune diseases than men [1, 2]. Moreover, the effectiveness of drug therapy as well as adverse drug reactions differ between men and women. However, evidence for sex differences in the incidence of adverse reactions to drugs is still limited. Early clinical trials on drugs were mainly performed in Caucasian young male participants because women were excluded due to hormonal fluctuations and the chance of being pregnant [3]. This also applied to preclinical trials in which the majority of animals were male [4]. Consequently, the majority of drugs that were marketed before the 1990s had only been studied in male animals and men. Today, these drugs are used on a large scale in both women and men. Between January 1st, 1997, and December 31st in 2000, the FDA withdrew 10 drugs from the market, eight of which because they posed a higher risk of adverse reactions in women than in men [5]. New trials are now incorporating more diverse participants due to the introduction of the ICH guideline in 1997, which recommends to include participants who are representative of the user population [6]. Meanwhile, information on drug safety in women is still limited especially in drugs marketed before the 1990s. Earlier research showed that women are 1.5–1.7 times more prone to develop ADRs than men [7]. Furthermore, the majority of the studies that showed that women are more at risk for ADRs than men are based on spontaneous reports of ADRs that can suffer from reporting bias between women and men [8–11]. An earlier study related the risk difference between women and men to the total number of hospital admissions and the total number of prescriptions; however, it was not possible to adjust for age and drug use on patient level due to the ecological design [12].

Our main objective was to identify sex differences in the type and number of severe ADRs that lead to hospital admission. In order to examine this, we investigated the number of ADR-associated hospital admissions over the years for both women and men, which drugs and ADRs caused most hospital admissions, and whether the associated risk for the most common drug-ADR combinations differed between women and men.

## Methods

### Data source

Data were obtained from the PHARMO Database Network. The PHARMO Database Network is a population-based network of electronic healthcare databases and combines anonymous data from different primary and secondary healthcare settings in the Netherlands. The longitudinal nature of the PHARMO Database Network system enables the follow-up of a well-defined population in the Netherlands for an average period of 12 years. Currently, the PHARMO Database Network covers over 6 million active persons out of 17 million inhabitants of the Netherlands. The population within the PHARMO Database Network comprises persons from locations all over the Netherlands. As described by Kuiper et al., this population corresponds in age and sex to the demographics of the total Dutch population [13]. For this study, the Out-patient Pharmacy Database and the Hospital Admission Database was used. The Out-Patient Pharmacy Database comprises GP or specialist prescribed drugs dispensed by the out-patient pharmacy. The dispensing records include information on the type of product, date, strength, dosage regimen, quantity, route of administration, prescriber specialty and costs. Drugs are coded according to the Anatomical Therapeutic Chemical (ATC) classification [14]. The Hospital Admission Database comprises hospital admissions for more than 24 h and admissions for less than 24 h for which a bed is required (i.e. in-patient records). The records include information on hospital admission and discharge dates, discharge diagnoses, procedures and treating specialism. Data from 2005 until the end of 2017 were used for this study. Diagnoses are coded with the International Classification of Diseases 9th revision (ICD-9) in the period 2005–2012 and according to the ICD 10th revision from January 1st, 2013, onwards.

### Study population

All hospital admissions between 1st of January 2005 up to 31st of December 2017 that were attributed to a drug were selected conform the ICD-9 and ICD-10 coding system of patients with available pharmacy data [see Additional files 1 and 2 for a detailed description and list of EY-codes].

Only patients with at least one dispensing of the drug that was reported as the suspected cause of the admission 3 months prior to the admission date were included, considering that the maximum duration of a prescription in the Netherlands is 3 months and that changes in drug use in the preceding 3 months were reported to be a predictor of hospitalizations [15].

Patients younger than 16 years old were excluded because of a low number of hospital admissions. All other patients were categorized in the following age categories: 16–55, 56–65, 66–75, 76–85 and > 85 years old.

### Outcomes

The first outcome was the number of ADR-associated hospital admissions for both women and men over the years. The second outcome was an overview of the drugs and ADRs causing hospital admissions for women and men. The final outcome was the relative risk for hospital admissions for women and men associated with the most common drug-ADR combinations.

### Data analysis

The number of ADR-associated hospital admissions was expressed as the percentage of the total number of hospital admissions within the PHARMO Database Network for both women and men. The most common drug groups coded as the probable cause of an ADR and the most common ADRs, both coded by medical coders, were assessed by (hospital) pharmacists and the supervising committee. For all drug-ADR combinations with at least 50 admissions for either women or men, age-adjusted odds ratios (ORs) with 95% confidence limits and *p* values were calculated for women versus men with respect to the total number of female and male users with a logistic regression model. Odds ratios were used as an estimate of the relative risk of a hospital admission due to a drug. Data analyses were performed using SAS Enterprise Guide (version 7.1) [16].

## Results

In the period between 2005 and 2017, 9,575,947 hospital admissions of patients aged 16 years and older (55.6% in women) were registered in the PHARMO Database Network. Of these admissions, 33,147 had an ICD code relating the admission to a drug (0.35 % of all hospital admissions). Within the ADR-related hospital admissions, 18,469 admissions concerned women (0.35 % of all hospital admissions in women and 55.7% of all ADR-associated admissions) and 14,678 admissions concerned men (0.35 % of all hospital admissions in men and 44.3% of all ADR-associated admissions). Women had a mean age of 72.1 years at admission which was statistically significantly higher than men who had a mean age of 71.3 years in ADR-related hospital admissions (Table 1).

**Table 1:** Age distribution of women and men with ADR-associated hospital admissions between the 1st of January 2005 up to the 31st of December 2017

		Women (18,469)	Men (14,678)
Mean age, years (SD)		72.1 (15.5)	71.3 (13.1)
Age categories, years, *n* (%)	16–55	2666 (14.4)	1650 (11.2)
	56–65	2460 (13.3)	2459 (16.8)
	66–75	3886 (21.0)	4183 (28.5)
	76–85	6070 (32.9)	4823 (32.9)
	> 85	3387 (18.3)	1563 (10.6)

**Fig. 1:**
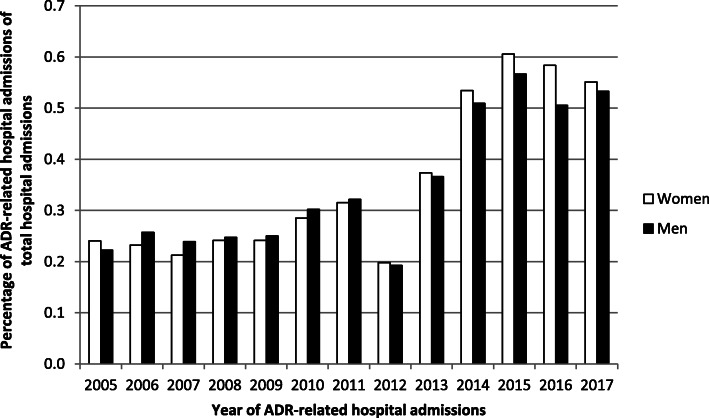
The percentage of ADR-related hospital admissions of the total number of hospital admissions. In total, there were 5,323,990 women and 4,251,957 men admitted

There was no difference between women and men in the proportions of ADR-related hospitalizations (Fig. 1). The number of ADR-associated hospital admissions increased over time, except for a lower number in 2012.

**Fig. 2:**
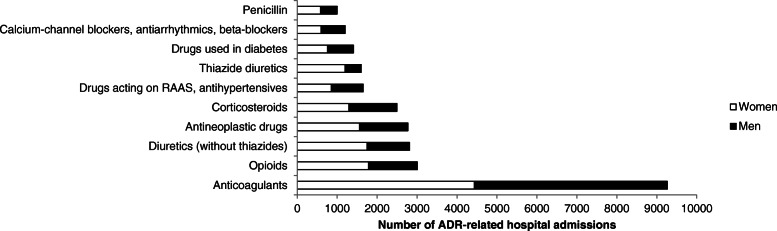
The number of ADR-related hospital admissions of women and men per drug group. These groups are the ten most frequently responsible drug groups causing hospital admissions

**Fig. 3:**
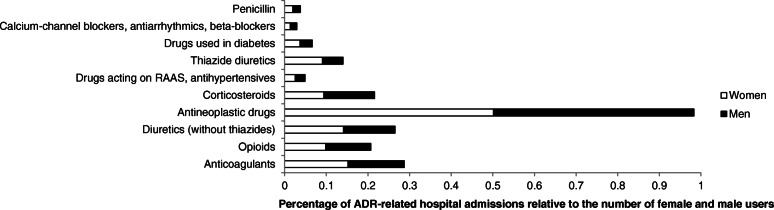
The percentage of ADR-related hospital admissions in women and men. The percentage is relative to the number of female and male users

The ADR-related hospital admissions were caused by 80 different drug groups and included 2213 different ADRs. The 10 drug groups with the highest number of ADR-related admissions are presented in Fig. 2. The percentage of ADR-related hospital admissions with respect to the number of female and male users are shown in Fig. 3. The 10 most frequent ADRs that were responsible for the admissions are shown in Table 2.

**Table 2:** The 10 most frequent ADRs responsible for hospital admissions for women and men

Adverse drug reaction	Total admissions (***n***)	Women (***n***)	Women (% of total female admissions)	Men (***n***)	Men (% of total male admissions)
Constipation	788	471	0.009	317	0.007
Hypo-osmolality and hyponatraemia	751	597	0.011	154	0.004
Haemorrhage, not elsewhere classified	650	365	0.007	285	0.007
Urinary tract infection, site not specified	625	422	0.008	203	0.005
Gastrointestinal haemorrhage, unspecified	606	290	0.005	316	0.007
Nausea and vomiting	575	392	0.007	183	0.004
Syncope and collapse	572	297	0.006	275	0.006
Pneumonia, unspecified	504	238	0.004	266	0.006
Drug-induced fever	492	264	0.005	228	0.005
Heart failure, unspecified	434	233	0.004	201	0.005

**Fig. 4:**
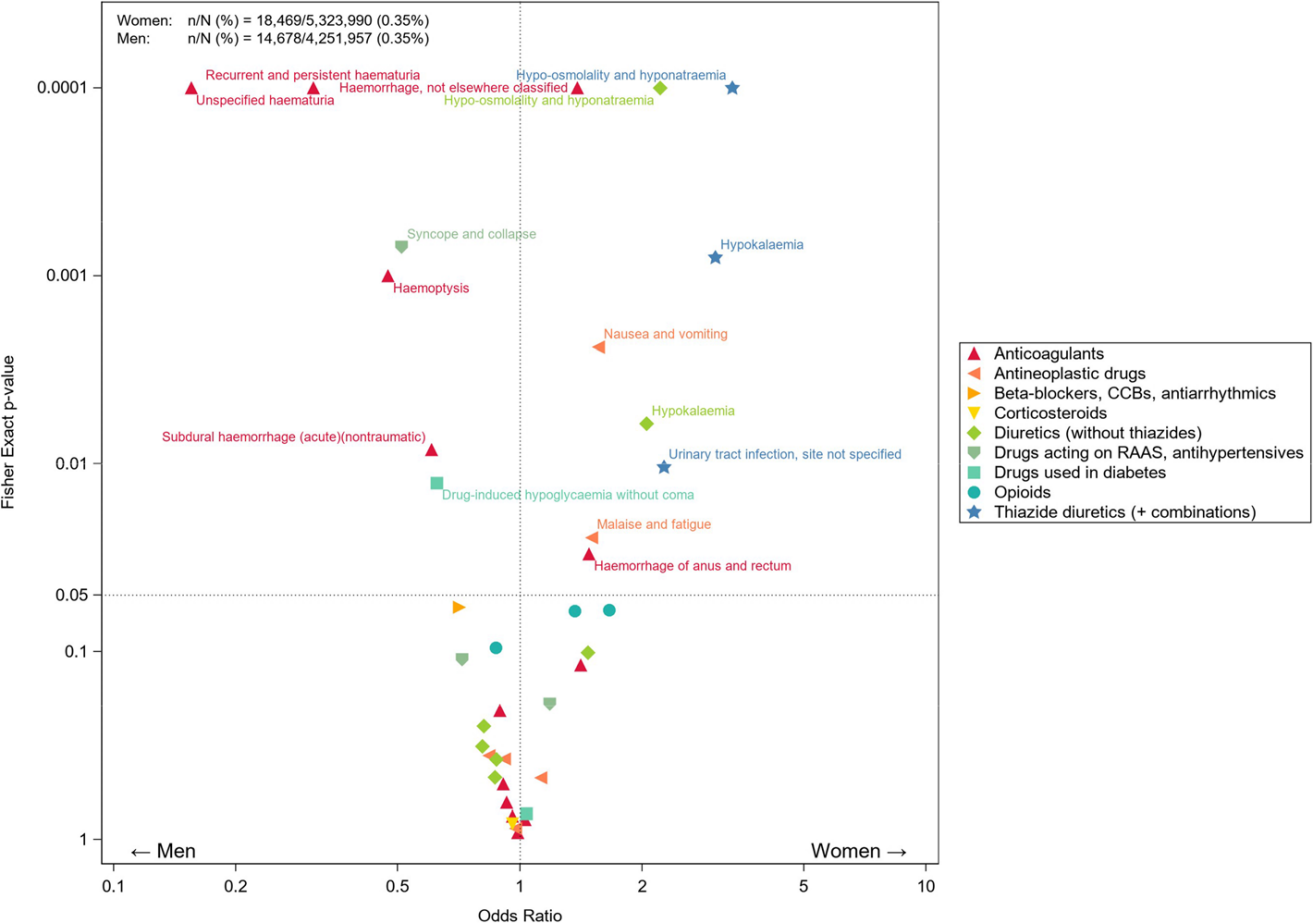
In total, 18,469 women (*n*) and 14,678 men (*n*) had an ADR-associated admission. This was 0.35% of the total number of admitted women (*N* = 5,323,990) and 0.35% of the total number of admitted men (*N* = 4,251,957). This figure shows the age-adjusted odds ratios (ORadj) for women versus men for drug-ADR combinations. All combinations with at least 50 admissions in either women or men are presented. ORs with a *p* value < 0.05 are statistically significant and labelled with the corresponding ADR [see Additional file 3 for all values]

There were 7797 unique drug-ADR combinations, 39 combinations had at least 50 hospital admissions in either women or men. In total, 9 drug groups were associated with 36 different adverse drug reactions. Figure 4 shows the age-adjusted ORs with corresponding *p* values of the drug-ADR combinations for women versus men. In 9 combinations, women were more at risk and in 6 combinations men were more at risk. Detailed results are shown in Additional file 3.

The most distinct differences in risk between women and men, as shown in Fig. 4, were seen in ADR-related hospitalizations due to anticoagulants. Anticoagulants were associated with a lower risk of a hospital admission with unspecified haematuria (ORadj 0.16; 95% CI 0.09, 0.34), recurrent and persistent haematuria (ORadj 0.31; 95% CI 0.21, 0.45), haemoptysis (ORadj 0.47, 95% CI 0.30, 0.74), subdural haemorrhage (ORadj 0.61; 95% CI 0.42, 0.88) in women and a higher risk of haemorrhage of anus and rectum (ORadj 1.48; 95% CI 1.04, 2.11) and haemorrhage, not elsewhere classified (ORadj 1.38; 95% CI 1.18, 1.62) in women.

Another drug group that showed large differences were diuretics which were associated with a higher risk of hospital admission due to hypokalaemia for women when using thiazides (ORadj 3.03; 95% CI 1.58, 5.79) and other diuretics (ORadj 2.05 95% CI 1.23, 3.42). Furthermore, women had an increased risk of admission due to hyponatraemia with thiazide use (ORadj 3.33, 95% CI 2.31, 4.81) and other diuretics (ORadj 2.21, 95% CI 1.63, 3.00). Thiazide diuretics also showed an increased risk of a hospital admission due to urinary tract infections (ORadj 2.26; 95% CI 1.21, 4.23) for women compared with men.

Antihypertensive drugs including drugs acting on the renin-angiotensin system showed a lower risk in women for hospital admissions due to syncope and collapse (ORadj 0.51; 0.35, 0.75) than in men. Antineoplastic drugs used by women resulted in a higher risk for hospital admissions due to malaise and fatigue (ORadj 1.50; 95% CI 1.05, 2.14) and due to nausea and vomiting (ORadj 1.56; 95% CI 1.17, 2.08). Hypoglycaemia as an ADR caused by drugs used in diabetes showed a statistically significantly lower risk in women (ORadj 0.63; 95% CI 0.43, 0.91) than in men.

## Discussion

Our study showed that 0.35% of all hospital admissions were coded as an ADR-related admission. There was no difference in the proportion of ADR-related hospital admissions between women and men.

There was a difference in risk for specific drug-ADR combinations; women were more at risk in 9 and men in 6 combinations. The most distinct differences were seen in ADRs due to anticoagulants and diuretics.

The 0.35% of ADR-related hospital admissions we found was much lower than the 5% mentioned in the literature [17]. Poor recognition and registration of ADRs as the cause of admission could explain this discrepancy. In addition, most of the studies into ADR-related hospital admissions consist of actively reported or actively registered ADRs at the time of admission resulting in a higher chance of recognition than in retrospective data [8, 9, 17–22]. We studied hospital admissions that are coded after discharge; therefore, our results are also influenced by the method and accuracy of coding by the hospitals. This problem has been described before [22]. Although this probably leads to underreporting, it will not influence differences in risk between women and men. The frequency of ADR-related hospital admissions increased over time for both women and men. This could be explained by changes in the methods of coding over time and the switch from the ICD-9 to ICD-10 coding system causing the low frequency in 2012. Furthermore, there has been more attention for ADRs as a cause of admission and for coding over the last few years as a measure of healthcare quality [23]. In addition, there was a difference in the number of participating hospitals over time and that might influence the increase in the number of ADR-related admissions due to differences in coding between hospitals. However, this will not influence differences between women and men.

Literature suggests that women tend to have a 1.5–1.7 times higher risk of developing ADRs [7]. Also, women report more ADRs than men; therefore, research into sex differences in ADRs is possibly subject to reporting bias caused by gender differences [8–11]. In contrast, we showed that there was no difference between women and men in the proportion of severe ADRs that were associated with hospital admissions.

Anticoagulants can cause, as a result of the mechanism of intended action, different types of haemorrhages. Rodenburg et al. showed that the risk of a hospital admission differs between women and men and for different types of bleeding [12]. We show similar results. In our study, men were more at risk for admission caused by unspecified, and recurrent and persistent haematuria, haemoptysis and subdural haemorrhage. The higher risk of haematuria might be explained by the development of prostate cancer [24]. Haemoptysis is a known complication of lung cancer which is more common in men and might explain the difference [25]. However, the incidence in women is increasing relative to the incidence in men [26]. If the haemoptysis admissions are due to lung cancer, the difference in risk will diminish over time. The risk of unspecified haemorrhage and haemorrhage of the anus and rectum was higher in women. Rectal bleeding is a known symptom of colorectal cancer. Interestingly, men more often have left-sided colorectal cancer which presents with rectal bleeding whereas women more often have right-sided colorectal cancer with symptoms such as anaemia and weight loss [27, 28]. Therefore, the higher risk of rectal bleeding in women is not explained by colorectal cancer. We did not find the difference in gastrointestinal haemorrhage that Rodenburg et al. found after adjustment for the number of prescriptions [12]. This might be the result of the introduction of the medical pharmaceutical decision rule on gastric protection for non-steroidal anti-inflammatory drugs, developed by the Royal Dutch Pharmacists Association and Health Base in 2013 [29]. Women might more often receive gastric protection for the prevention of gastric complications, as recommended by the Dutch guideline [30], due to more frequent use of interacting drugs such as selective serotonin reuptake inhibitors (SSRIs).

An increased risk of women to be admitted due to electrolyte disturbances, hyponatraemia and hypokalaemia, when using diuretics was another result Rodenburg et al. found [12]. We showed similar results in both thiazides and other diuretics. Women might be more at risk of hyponatraemia because sex hormones influence the regulation of arginine vasopressin (AVP) resulting in a higher renal sodium excretion in women as shown in a study on sex differences in the regulation of AVP during hypotonic saline infusion [31]. They suggested that testosterone has a greater influence on the renal sodium excretion than oestrogen because the excretion did not differ between phases of the menstrual cycle. The potentially higher risk of hyponatremia in general along with the use of thiazide diuretics, which are known to cause electrolyte disturbances because of their mechanism of action [32], might explain the higher risk in women compared with men. Another result we found was a higher risk in women to develop urinary tract infections (UTIs) when using thiazide diuretics. Diuretics have been described to cause lower urinary tract symptoms before but the underlying mechanism is still unknown [33]. However, the difference in risk we found between women and men might be explained by the higher incidence of UTIs in women compared with men [34]. It is possible that women are more frequently tested for UTIs than men when admitted with unexplained symptoms such as incontinence, nocturnal voiding, confusion and malaise and elder women have a higher risk of asymptomatic bacteriuria than men [35]. Also, the admissions could be the result of dehydration due to the urinary tract infection and concomitant use of thiazides.

A higher risk for men than women of hospital admissions due to syncope and collapse when using antihypertensive drugs and drugs acting on the renin-angiotensin system was also found by Rodenburg et al. [12]. We found this association within a larger drug group due to differences in coding between ICD-9 and ICD-10 and therefore included ATC code C09 (drugs acting on the renin-angiotensin system) in addition to ATC code C02 (antihypertensive drugs). Sex differences in the renin-angiotensin system have been described, but show contrasting results. It has been suggested that angiotensin-converting enzyme (ACE) inhibitors have a larger effect on the reduction of blood pressure in male rats and male rats have a higher ACE2 activity [36]. A recent study suggested that women with heart failure (HF) reach the same treatment effects (i.e. relative risk of mortality or hospital admission for HF) with lower doses of RAAS blockers than men, and do not benefit from up-titrating to guideline-recommended doses [37]. This means that the underlying pathophysiology of HF may interfere with treatment effects in a sex-specific manner. Further research could give more insight into which drugs are responsible for this reaction and if the indication for treatment is an additional risk factor.

Antineoplastic drugs posed a higher risk in women for malaise, fatigue, nausea and vomiting. All these ADRs are mentioned in the literature to occur more frequently in women [8]. We now confirmed that not only in spontaneous reporting systems there is a higher risk for women but also in the systematic recorded cases we studied.

Rodenburg et al. showed that men have a higher risk to be admitted due to drugs used in diabetes causing hypoglycaemia resulting into coma [12]. We saw a higher risk for men for hypoglycaemia without coma but not in admission for hypoglycaemia with coma.

The sex differences in ADR-related hospital admissions described above can be due to several differences between women and men in pharmacokinetics (PK) and pharmacodynamics (PD) [38–41]. However, more research is needed to determine whether the underlying mechanisms can be explained by PK and/or PD differences.

One of the strengths of our study was the minimization of reporting bias by investigating only ADR–related hospitalizations. These ADRs are so severe that it was expected that women and men are equally admitted to a hospital. This could explain why we do not see a difference in the number of ADR-related hospital admissions with respect to the total number of admissions for women and men. Another strength of this study was the availability of individual patient information, which enables to adjust for age.

A limitation of this study was that the total number of users is not the total number of patients at risk. This is due to changes in data coverage. The hospitals that contribute are different over the years. Whereas the pharmacy data is available from a specific number and region. It is possible that pharmacy data of the patient was available; however, the patient was admitted to a hospital that was not included. This might influence the number of admissions; however, this will not result in differences between women and men. Another limitation was the unavailability of inpatient use of antineoplastic drugs. Antineoplastic drugs show, relative to the number of users, the highest number of ADR-related hospital admissions. However, this was only adjusted for the number of antineoplastic drugs that are used in the outpatient setting. Parenteral administrations or other administrations of antineoplastic drugs in the hospital are not registered in the Outpatient Pharmacy database. This will not have an effect on the difference between women and men but can influence the proportion of ADR-related hospital admissions compared with other drug groups. Several cardiovascular drugs were coded differently in ICD-9 and ICD-10 and therefore combined into one large group including ATC codes C01B (antiarrhythmic drugs), C07AA, C07AB, C07B, C07C (beta-blocking agents and combinations) and C08 (calcium channel blockers). There could be differences in risk for each of the subgroups; however, we are not able to identify it by the method of coding. Another limitation was that we were not able to adjust for co-morbidities. We did compare the chronic disease scores for all patients with an ADR-associated hospital admission. Women had a slightly lower mean chronic disease score than men, which should be taken into account in future research. Furthermore, we were not able to adjust for potential differences in drug dose and weight that can differ between women and men. Although we are unaware of sex-specific dosing guidelines in the Netherlands, except for drugs dosed on body weight or body surface area resulting in a more sex-specific dose, ADRs cannot only be explained by sex differences in weight [42]. Also, we were not able to measure the adherence to the treatment that might differ between women and men. Several studies on sex and gender differences in adherence show that women are less adherent than men [43–45], although no differences were seen in antihypertensive drug users [46].

### Perspectives and significance

We show that there was no difference in the proportion of ADR-related hospital admissions between women and men. However, there were differences in the risk of hospital admission in specific drug-ADR combinations. The most substantial differences were seen in ADRs due to anticoagulants and diuretics. Further research is needed to adjust for potential confounding factors that might have influenced the results, for example drug dose, other drugs in use and comorbidities. In addition, research into PK and PD is necessary to investigate whether these sex differences in ADRs are predicted by PK or PD. This would provide information for personalized medicine in the future.

## Supplementary Information

**Additional file 1.**

**Additional file 2.**

**Additional file 3.**

## Data Availability

The data that support the findings of this study are available from the PHARMO Institute but restrictions apply to the availability of these data, which were used under licence for the current study, and so are not publicly available. Data are however available from the authors upon reasonable request and with permission of the PHARMO Institute.

## References

[1] Vetvik KG, MacGregor EA (2017). Sex differences in the epidemiology, clinical features, and pathophysiology of migraine. Lancet Neurol..

[2] Myasoedova E, Crowson CS, Kremers HM, Therneau TM, Gabriel SE (2010). Is the incidence of rheumatoid arthritis rising?: results from Olmsted County, Minnesota, 1955-2007. Arthritis Rheum..

[3] Schiebinger L (2003). Women's health and clinical trials. J Clin Invest..

[4] Beery AK, Zucker I (2011). Sex bias in neuroscience and biomedical research. Neurosci Biobehav Rev..

[5] Office USGA (2001). Drug Safety: Most Drugs Withdrawn in Recent Years Had Greater Health Risks for Women.

[6] Group" IEW (1997). General Considerations for Clinical Trials.

[7] Martin RM, Biswas PN, Freemantle SN, Pearce GL, Mann RD (1998). Age and sex distribution of suspected adverse drug reactions to newly marketed drugs in general practice in England: analysis of 48 cohort studies. Br J Clin Pharmacol..

[8] de Vries ST, Denig P, Ekhart C, Burgers JS, Kleefstra N, Mol PGM (2019). Sex differences in adverse drug reactions reported to the National Pharmacovigilance Centre in the Netherlands: An explorative observational study. Br J Clin Pharmacol..

[9] Watson S, Caster O, Rochon PA, den Ruijter H (2019). Reported adverse drug reactions in women and men: Aggregated evidence from globally collected individual case reports during half a century. EClinicalMedicine..

[10] Zopf Y, Rabe C, Neubert A, Gassmann KG, Rascher W, Hahn EG (2008). Women encounter ADRs more often than do men. Eur J Clin Pharmacol..

[11] Rademaker M (2001). Do Women Have More Adverse Drug Reactions?. Am J Clin Dermatol..

[12] Rodenburg EM, Stricker BH, Visser LE (2011). Sex-related differences in hospital admissions attributed to adverse drug reactions in the Netherlands. Br J Clin Pharmacol..

[13] Kuiper JG, Bakker M, Penning-van Beest FJA, Herings RMC (2020). Existing Data Sources for Clinical Epidemiology: The PHARMO Database Network. Clin Epidemiol..

[14] WHO. 05-12-2019 [cited 2020 02-03-2020]; Available from: https://www.whocc.no/. Accessed 2 Mar 2020.

[15] Parameswaran Nair N, Chalmers L, Connolly M, Bereznicki BJ, Peterson GM, Curtain C, et al. Prediction of Hospitalization due to Adverse Drug Reactions in Elderly Community-Dwelling Patients (The PADR-EC Score). PLoS One. 2016;11(10):e0165757. 10.1371/journal.pone.0165757. 10.1371/journal.pone.0165757 PMC5087856 27798708

[16] Matange S. CTSPedia Clinical Graphs - Volcano Plot. 2016 23-05-2016 [cited 2021 16-03-2021]; Available from: https://blogs.sas.com/content/graphicallyspeaking/2016/05/23/ctspedia-clinical-graphs-volcano-plot/. Accessed 16 Mar 2021.

[17] Leendertse AJ, Egberts ACG, Stoker LJ, van den Bemt PMLA (2008). Frequency of and Risk Factors for Preventable Medication-Related Hospital Admissions in the Netherlands. Arch Intern Med..

[18] Alayed N, Alkhalifah B, Alharbi M, Alwohaibi N, Farooqui M (2019). Adverse Drug Reaction (ADR) as a Cause of Hospitalization at a Government Hospital in Saudi Arabia: A Prospective Observational Study. Curr Drug Saf..

[19] Light KP, Lovell AT, Butt H, Fauvel NJ, Holdcroft A (2006). Adverse effects of neuromuscular blocking agents based on yellow card reporting in the U.K.: are there differences between males and females?. Pharmacoepidemiol Drug Saf..

[20] Onder G, Pedone C, Landi F, Cesari M, Vedona CD, Bernabei R (2002). Adverse Drug Reactions as Cause of Hospital Admissions: Results from the Italian Group of Pharmacoepidemiology in the Elderly (GIFA). J Am Geriatr Soc..

[21] Yu Y, Chen J, Li D, Wang L, Wang W, Liu H (2016). Systematic Analysis of Adverse Event Reports for Sex Differences in Adverse Drug Events. Sci Rep..

[22] Leendertse AJ, Visser D, Egberts ACG, van den Bemt PMLA (2010). The Relationship Between Study Characteristics and the Prevalence of Medication-Related Hospitalizations A Literature Review and Novel Analysis. Drug Saf..

[23] van der Laan J, de Bruin A, van den Akker-Ploemacher J, Penning C, Pijpers F (2015). HSMR 2014: Methodological report.

[24] Merriel SWD, Funston G, Hamilton W (2018). Prostate Cancer in Primary Care. Adv Ther..

[25] Hamilton W, Sharp D (2004). Diagnosis of lung cancer in primary care: a structured review. Fam Pract..

[26] Lu T, Yang X, Huang Y, Zhao M, Li M, Ma K, Yin J, Zhan C, Wang Q (2019). Trends in the incidence, treatment, and survival of patients with lung cancer in the last four decades. Cancer Manag Res..

[27] Kim SE, Paik HY, Yoon H, Lee JE, Kim N, Sung MK (2015). Sex- and gender-specific disparities in colorectal cancer risk. World J Gastroenterol..

[28] Benedix F, Kube R, Meyer F, Schmidt U, Gastinger I, Lippert H, Colon/Rectum Carcinomas (Primary Tumor) Study Group (2010). Comparison of 17,641 patients with right- and left-sided colon cancer: differences in epidemiology, perioperative course, histology, and survival. Dis Colon Rectum..

[29] KNMP, Base H, MFB E. Maagbescherming bij NSAID-gebruik (gastric protection when NSAIDs are used); 2013.

[30] De Jongh E, De Wit NJ, Numans ME, Smeink P, Van der Weele GM, Wesseler GH. Preventie van maagcomplicaties door medicijngebruik / Prevention of gastric complications due to drug use. 2021 March 2021 [cited 2021 16-03-2021]; Available from: https://richtlijnen.nhg.org/behandelrichtlijnen/preventie-van-maagcomplicaties-door-medicijngebruik#volledige-tekst. Accessed 16 Mar 2021.

[31] Stachenfeld NS, Splenser AE, Calzone WL, Taylor MP, Keefe DL (2001). Genome and Hormones: Gender Differences in Physiology Selected Contribution: Sex differences in osmotic regulation of AVP and renal sodium handling. J Appl Physiol..

[32] Hwang KS, Kim GH (2010). Thiazide-induced hyponatremia. Electrolyte Blood Press..

[33] Hall SA, Chiu GR, Kaufman DW, Wittert GA, Link CL, McKinlay JB (2012). Commonly used antihypertensives and lower urinary tract symptoms: results from the Boston Area Community Health (BACH) Survey. BJU Int..

[34] Bouma M, Geerlings SE, Klinkhamer S, Knottnerus BJ, Platteel TN, Reuland EA (2020). Urineweginfecties (urinary tract infections). Nederlands Huisartsen Genootschap (The Dutch College of General Practitioners).

[35] Biggel M, Heytens S, Latour K, Bruyndonckx R, Goossens H, Moons P (2019). Asymptomatic bacteriuria in older adults: the most fragile women are prone to long-term colonization. BMC Geriatr..

[36] Ahmed S, Hu R, Leete J, Layton AT (2019). Understanding sex differences in long-term blood pressure regulation: insights from experimental studies and computational modeling. Am J Physiol Heart Circ Physiol..

[37] Santema BT, Ouwerkerk W, Tromp J, Sama IE, Ravera A, Regitz-Zagrosek V, et al. Identifying optimal doses of heart failure medications in men compared with women: a prospective, observational, cohort study. Lancet. 2019;394:1254–63. 10.1016/S0140-6736(19)31792-1. 10.1016/S0140-6736(19)31792-1 31447116

[38] Whitley HP, Lindsey W (2009). Sex-Based Differences in Drug Activity. Am Fam Physician..

[39] Islam MM, Iqbal U, Walther BA, Nguyen PA, Li YJ, Dubey NK (2017). Gender-based personalized pharmacotherapy: a systematic review. Arch Gynecol Obstet..

[40] Handbook of Experimental Pharmacology. Sex and Gender Differences in Pharmacology. Volume 214. Heidelberg: Springer- Verlag; 2012. p. 1–587.

[41] Farkouh A, Riedl T, Gottardi R, Czejka M, Kautzky-Willer A. Sex-Related Differences in Pharmacokinetics and Pharmacodynamics of Frequently Prescribed Drugs: A Review of the Literature. Adv Ther. 2019;37:644–55. 10.1007/s12325-019-01201-3. 10.1007/s12325-019-01201-3 31873866

[42] Zucker I, Prendergast BJ (2020). Sex differences in pharmacokinetics predict adverse drug reactions in women. Biol Sex Differ..

[43] Manteuffel M, Williams S, Chen W, Verbrugge RR, Pittman DG, Steinkellner A (2014). Influence of patient sex and gender on medication use, adherence, and prescribing alignment with guidelines. J Womens Health (Larchmt)..

[44] Eindhoven DC, Hilt AD, Zwaan TC, Schalij MJ, Borleffs CJW (2018). Age and gender differences in medical adherence after myocardial infarction: Women do not receive optimal treatment - The Netherlands claims database. Eur J Prev Cardiol..

[45] Granger BB, Ekman I, Granger CB, Ostergren J, Olofsson B, Michelson E, McMurray JJV, Yusuf S, Pfeffer MA, Swedberg K (2009). Adherence to medication according to sex and age in the CHARM programme. Eur J Heart Fail..

[46] Biffi A, Rea F, Iannaccone T, Filippelli A, Mancia G, Corrao G (2020). Sex differences in the adherence of antihypertensive drugs: a systematic review with meta-analyses. BMJ Open..

