# Supplementary material for: Sex differences associated with adverse drug reactions resulting in hospital admissions
Source: Biol Sex Differ. 2021 May 3;12:34. doi: 10.1186/s13293-021-00377-0 (PMC8091530; doi:10.1186/s13293-021-00377-0)
Supplement: Supplementary file 1 — Additional file 1. [file 13293_2021_377_MOESM1_ESM.docx]

**Additional file 1**

Hospital admissions were coded according the ICD 9 or ICD 10 coding system. The primary discharge diagnosis was the adverse drug reaction and the secondary discharge diagnosis with E93-E94 (ICD-9) and Y40-Y59 (ICD-10) codes identify the drug. The E93-E94 (ICD-9) and Y40-Y59 (ICD-10) codes were recoded into a new code (EY-code) to combine the codes that overlapped due to differences in categorization between ICD9 and ICD 10. The table shows the new categorization with the corresponding ATC codes. All hospital discharges that were coded with the ICD 9 system were recoded into ICD 10 codes. The most common drug-ADR combinations were assessed by (hospital)pharmacists and the supervising committee. Drug-ADR combinations that were assessed to be unlikely or unspecified, for example drugs that are administered for treatment of the diagnosis, admissions due to an unspecified traffic incident and admissions with an unspecified adverse effect, were excluded.
