# Supplementary material for: Sex differences associated with adverse drug reactions resulting in hospital admissions
Source: Biol Sex Differ. 2021 May 3;12:34. doi: 10.1186/s13293-021-00377-0 (PMC8091530; doi:10.1186/s13293-021-00377-0)
Supplement: Supplementary file 3 — Additional file 3. [file 13293_2021_377_MOESM3_ESM.docx]

**Additional file 3**

The following tables contain the number of women and men and ORs for the specific drug-ADR combinations. The age-adjusted ORs (OR adj) are the values shown in figure 4.

| EY020 - Corticosteroids |  |  |  |  |
| --- | --- | --- | --- | --- |
| **Adverse drug reaction** | **Women (N)** | **Men (N)** | **OR (95% CI)** | **OR adj (95% CI)** |
| Chronic obstructive pulmonary disease with acute lower respiratory infection | 59 | 46 | 0.92 (0.62, 1.35) | 0.96 (0.65, 1.41) |

| EY023 - Drugs used in diabetes |  |  |  |  |
| --- | --- | --- | --- | --- |
| **Adverse drug reaction** | **Women (N)** | **Men (N)** | **OR (95% CI)** | **OR adj (95% CI)** |
| Drug-induced hypoglycaemia without coma | 51 | 68 | 0.81 (0.56, 1.17) | 0.63 (0.43, 0.91) |
| Hypoglycaemia, unspecified | 204 | 176 | 1.25 (1.02, 1.53) | 1.04 (0.85, 1.27) |
| EY031 - Antineoplastic drugs |  |  |  |  |
| **Adverse drug reaction** | **Women (N)** | **Men (N)** | **OR (95% CI)** | **OR adj (95% CI)** |
| Toxic gastroenteritis and colitis | 55 | 56 | 0.80 (0.55, 1.15) | 0.84 (0.58, 1.22) |
| Drug-induced fever | 216 | 197 | 0.89 (0.73, 1.08) | 0.92 (0.76, 1.11) |
| Allergic and dietetic gastroenteritis and colitis | 93 | 82 | 0.92 (0.68, 1.24) | 0.98 (0.73, 1.31) |
| Agranulocytosis | 88 | 66 | 1.08 (0.78, 1.49) | 1.13 (0.82, 1.55) |
| Malaise and fatigue | 84 | 48 | 1.42 (0.99, 2.02) | 1.50 (1.05, 2.14) |
| Nausea and vomiting | 133 | 72 | 1.49 (1.12, 1.99) | 1.56 (1.17, 2.08) |

| EY041 - Anticoagulants |  |  |  |  |
| --- | --- | --- | --- | --- |
| **Adverse drug reaction** | **Women (N)** | **Men (N)** | **OR (95% CI)** | **OR adj (95% CI)** |
| Unspecified haematuria | 17 | 103 | 0.20 (0.12, 0.34) | 0.16 (0.09, 0.26) |
| Recurrent and persistent haematuria | 38 | 122 | 0.38 (0.27, 0.55) | 0.31 (0.21, 0.45) |
| Haemoptysis | 28 | 70 | 0.49 (0.32, 0.76) | 0.47 (0.30, 0.74) |
| Subdural haemorrhage (acute)(nontraumatic) | 44 | 81 | 0.67 (0.46, 0.97) | 0.61 (0.42, 0.88) |
| Gastrointestinal haemorrhage, unspecified | 246 | 279 | 1.09 (0.91, 1.29) | 0.89 (0.75, 1.06) |
| Haemorrhage and haematoma complicating a procedure, not elsewhere classified | 86 | 113 | 0.94 (0.71, 1.24) | 0.91 (0.68, 1.21) |
| Intracerebral haemorrhage in hemisphere, subcortical | 79 | 87 | 1.12 (0.82, 1.52) | 0.93 (0.68, 1.27) |
| Epistaxis | 114 | 119 | 1.18 (0.91, 1.53) | 0.96 (0.74, 1.25) |
| Melaena | 120 | 123 | 1.20 (0.93, 1.55) | 0.99 (0.76, 1.28) |
| Haemorrhagic disorder due to circulating anticoagulants | 207 | 209 | 1.22 (1.01, 1.48) | 1.03 (0.85, 1.25) |
| Haemorrhage, not elsewhere classified | 362 | 281 | 1.59 (1.36, 1.85) | 1.38 (1.18, 1.62) |
| Iron deficiency anaemia secondary to blood loss (chronic) | 52 | 37 | 1.73 (1.14, 2.64) | 1.41 (0.92, 2.17) |
| Haemorrhage of anus and rectum | 77 | 54 | 1.76 (1.24, 2.49) | 1.48 (1.04, 2.11) |

| EY050 - Opioids |  |  |  |  |
| --- | --- | --- | --- | --- |
| **Adverse drug reaction** | **Women (N)** | **Men (N)** | **OR (95% CI)** | **OR adj (95% CI)** |
| Constipation | 362 | 255 | 0.89 (0.75, 1.04) | 0.87 (0.74, 1.02) |
| Nausea and vomiting | 123 | 52 | 1.47 (1.07, 2.04) | 1.37 (0.99, 1.89) |
| Poisoning by narcotics and psychodysleptics [hallucinogens] (Other opioids) | 53 | 19 | 1.74 (1.03, 2.94) | 1.66 (0.98, 2.81) |

| EY121 - Calcium-channel blockers, antiarrhythmics, beta-blockers |  |  |  |  |
| --- | --- | --- | --- | --- |
| **Adverse drug reaction** | **Women (N)** | **Men (N)** | **OR (95% CI)** | **OR adj (95% CI)** |
| Bradycardia, unspecified | 61 | 63 | 0.85 (0.60, 1.21) | 0.71 (0.50, 1.01) |

| EY123 - Drugs acting on renin-angiotensin system, antihypertensives |  |  |  |  |
| --- | --- | --- | --- | --- |
| **Adverse drug reaction** | **Women (N)** | **Men (N)** | **OR (95% CI)** | **OR adj (95% CI)** |
| Syncope and collapse | 44 | 66 | 0.67 (0.46, 0.98) | 0.51 (0.35, 0.75) |
| Hypotension due to drugs | 45 | 53 | 0.85 (0.57, 1.27) | 0.72 (0.48, 1.08) |
| Angioneurotic oedema | 139 | 114 | 1.22 (0.95, 1.56) | 1.18 (0.92, 1.52) |

| EY141 - Low ceiling diuretics (without thiazides), high ceiling diuretics, potassium sparing diuretics | | | | |
| --- | --- | --- | --- | --- |
| **Adverse drug reaction** | **Women (N)** | **Men (N)** | **OR (95% CI)** | **OR adj (95% CI)** |
| Volume depletion | 106 | 77 | 0.95 (0.71, 1.27) | 0.88 (0.65, 1.18) |
| Acute renal failure, unspecified | 57 | 27 | 1.46 (0.92, 2.30) | 1.47 (0.93, 2.33) |
| Hypokalaemia | 57 | 20 | 1.97 (1.18, 3.27) | 2.05 (1.23, 3.42) |
| Hypo-osmolality and hyponatraemia | 184 | 54 | 2.35 (1.73, 3.18) | 2.21 (1.63, 3.00) |
| Left ventricular failure | 50 | 40 | 0.86 (0.57, 1.31) | 0.81 (0.53, 1.23) |
| Heart failure, unspecified | 75 | 56 | 0.92 (0.65, 1.31) | 0.81 (0.57, 1.16) |
| Congestive heart failure | 64 | 48 | 0.92 (0.63, 1.34) | 0.87 (0.60, 1.27) |

| EY143 - Thiazide diuretics (+ combinations) |  |  |  |  |
| --- | --- | --- | --- | --- |
| **Adverse drug reaction** | **Women (N)** | **Men (N)** | **OR (95% CI)** | **OR adj (95% CI)** |
| Hypokalaemia | 58 | 11 | 3.22 (1.69, 6.13) | 3.03 (1.58, 5.79) |
| Hypo-osmolality and hyponatraemia | 224 | 33 | 4.15 (2.88, 5.98) | 3.33 (2.31, 4.81) |
| Urinary tract infection, site not specified | 59 | 12 | 3.00 (1.62, 5.59) | 2.26 (1.21, 4.23) |
